# Preferences for mode of delivery in nulliparous Argentinean women: a qualitative study

**DOI:** 10.1186/1742-4755-10-2

**Published:** 2013-01-14

**Authors:** Nancy H Liu, Agustina Mazzoni, Nina Zamberlin, Mercedes Colomar, Olivia H Chang, Lila Arnaud, Fernando Althabe, José M Belizán

**Affiliations:** 1 Department of Psychiatry-SFGH, University of California, San Francisco, Suite 7M. 1001 Potrero Avenue, San Francisco, CA, 94110, USA; 2 Institute for Clinical Effectiveness and Health Policy (IECS), Dr. Emilio Ravignani 2024. C1414CPV, Buenos Aires, Argentina; 3 Center for the Study of State and Society (CEDES), Sanchez de Bustamante 27. C1173AAA, Buenos Aires, Argentina; 4 Clinical and Epidemiological Research Unit (UNICEM), Hospital de Clinicas, Avda. Italia s/n, Montevideo, Uruguay; 5 Tulane University School of Medicine, 1430 Tulane Avenue, New Orleans, Louisiana, 70112, USA; 6 Tulane University School of Public Health and Tropical Medicine, 1440 Canal St, New Orleans, LA, 70112, USA

**Keywords:** Cesarean section, Women’s preferences, Mode of delivery, Qualitative

## Abstract

**Background:**

Over the last three decades, cesarean section (CS) rates have been rising around the world despite no associated improvement in maternal and perinatal mortality and morbidity. The role of women’s preferences for mode of delivery in contributing to the high CS rate remains controversial; however these preferences are difficult to assess, as they are influenced by culture, knowledge of risk and benefits, and personal and social factors. In this qualitative study, our objective was to understand women’s preferences and motivational factors for mode of delivery. This information will inform the development and design of an assessment aimed at understanding the role of the women’s preferences for mode of delivery.

**Methods:**

We conducted 4 focus group discussions (FGDs) and 12 in-depth interviews with pregnant women in Buenos Aires, Argentina in 4 large non-public and public hospitals. Our sample included 29 nulliparous pregnant women aged 18–35 years old, with single pregnancies over 32 weeks of gestational age, without pregnancies resulting from assisted fertility, without known pre-existing medical illness or diseases diagnosed during pregnancy, without an indication of elective cesarean section, and who are not health professionals. FGDs and interviews followed a pre-designed guide based on the health belief model and social cognitive theory of health decisions and behaviors.

**Results:**

Most of the women preferred vaginal delivery (VD) due to cultural, personal, and social factors. VD was viewed as normal, healthy, and a natural rite of passage from womanhood to motherhood. Pain associated with vaginal delivery was viewed positively. In contrast, women viewed CS as a medical decision and often deferred decisions to medical staff in the presence of medical indication.

**Conclusions:**

These findings converge with quantitative and qualitative studies showing that women prefer towards VD for various cultural, personal and social reasons. Actual CS rates appear to diverge from women’s preferences and reasons are discussed.

## Background

Over the last three decades, cesarean section (CS) rates have been rising around the world [1-3]. Fifteen countries with around 12 million births per year have CS rates over 30% [4]. In Latin America, 9 of the 12 countries have rates over the 15% limit recommended by the World Health Organization [2,5]. CS rates continue to rise despite no associated improvement in maternal and perinatal mortality and morbidity; rather, it can increase the risk of complications, such as maternal mortality, urinary tract injury, and hysterectomy [3,6-8].

A major obstacle to understanding the role of women's preferences for mode of delivery in the rise of CS rates is the lack of a standardized instrument. The role of women’s preferences for mode of delivery and decision-making remains a controversial factor contributing to the high rate of CS. This has neither been verified nor refuted due to the difficulty with measuring this construct. Preferences are influenced by culture, knowledge of risk and benefits, and personal and social factors. Reasons for women’s preferences range from perceived ease of recovery and need to return to family responsibilities to concerns about the safety of the baby [9-15]. Until a standardized assessment is available, our understanding of the role of women’s preferences is limited. Researchers have called for further qualitative research investigating the influence of obstetric, personal and social factors on women’s views of vaginal and cesarean birth [16].

We aim to understand the role of the women’s preferences in CS. In this qualitative study, our objective was to understand women’s preferences and motivational factors for mode of delivery to improve understanding about the personal and social factors that contribute to women’s preferences. Moreover, qualitative research provides the language and value-laden vocabulary used by women which may shed further insight on women’s preferences for the two modes of delivery. This information will be used to design an assessment of women’s preferences for mode of delivery.

## Methods

We conducted focus group discussions (FGDs) and in-depth interviews with pregnant women in Buenos Aires, Argentina including a convenience sample of 2 public and 2 non-public hospitals willing to participate in our study.

The Healthcare System in Argentina is composed of two sectors: a public sector, and a non-public sector. The public sector depends on the Ministry of Health and its beneficiaries include mostly persons without health insurance, usually from lower socio-economic backgrounds having no charge for their assistance. This sector assists 55% of the total deliveries of the country. The remaining 45% of the deliveries are assisted in non-public hospitals with a private administration having a charge to the assistance. In non-public hospitals around 90% of women have some kind of insurance to afford the charge of their deliveries. One is the social security sector grounded on the principle of social insurance, which requires all employers and employees to make contributions to a trust fund. The other includes different types of pre-paid health insurance packages and their beneficiaries are typically persons from high socio-economic backgrounds.

A purposive sampling strategy was used based on the following predefined criteria: 1) nulliparous pregnant women; 2) 18–35 years old; 3) with single pregnancies over 32 weeks of gestational age; 4) without pregnancies resulting from assisted fertility; 5) without known pre-existing medical illness or diseases diagnosed during pregnancy; 6) without an indication of elective CS; 7) have plans to deliver at a hospital participating in the study; and 8) are not health professionals.

Four FGDs and 12 in-depth interviews were conducted in Spanish. Public sector FGD were conducted in January 2009 in public sector, while non-public sector FGDs were conducted in June 2009. In-depth interviews were conducted between August and September 2010. We completed 2 public sector FGDs (7 women in the first group and 8 women in the second group) and 7 public sector interviews. We completed 2 non-public sector FGDs (7 women each) and 5 non-public sector interviews. A total of 29 women participated (15 women of the public sector and 14 of the non-public sector).

Providers collaborating with the study, approach women during the antenatal visits and made an evaluation about the eligibility criteria. Women having the eligible criteria were invited to participate. All participants provided informed consent and were reimbursed for transportation costs. All FGDs and interviews were audio-recorded, transcribed and analyzed by a sociologist with extensive experience with qualitative research methods in sexual and reproductive health.

FGDs and interviews followed a pre-designed guide based on a theoretical model of health decisions and behaviors [17,18]. Discussion topics were organized according to five domains: a) perceived outcomes of each mode of delivery, b) relevant referents of the decision, c) perceived barriers and facilitators to choosing each mode of delivery, d) characteristics, qualities and attributes of persons choosing each mode of delivery, and e) cultural factors. Transcripts were analyzed in Spanish and translated into English for the purposes of reporting the results. Results were analyzed by an expert in qualitative research without the use of qualitative software.

Approval for the study was obtained from the Tulane University Office of Research Protection IRB in the USA and the Medical Education and Clinical Investigation Center IRB in Argentina.

## Results

Characteristics of participants are shown in Table 1. Public sector women were generally younger, had lower levels of education, tended to live with extended families, and had less health care coverage than those who came from the non-public sector.

**Table 1:** Participant socio-demographic characteristics by sector

	**Public N= 15**	**Non-public N=14**
Age in years - Mean (SD)	20.4 (2.45)	33.1 (1.94)
Married (%)	75	100
Secondary school completed (%)^3^	50	100
Live within extended families (%)	40	0
Health care coverage (%)	20	100

^3^Extended families mean with family members other than their partners.

Our first aim was to understand the language and value-laden vocabulary used by women to refer to the two modes of delivery. Women used the word “normal” and “natural” delivery to refer to vaginal delivery (VD), and “cesarean” to refer to the CS. This terminology was shared by women from both the public and non-public sectors.

### Pain

The ideal delivery situation among our sample in both non-public and public sectors was described as a VD with short labor and a quick ending where pain could be controlled and suffering limited. Women wanted to reduce pain as much as possible but also wanted to be able to “feel childbirth.” The ideal subjective experience at the moment of delivery included the following characteristics: relaxed, in control of the process, accompanied by a partner or someone else in the delivery room and with immediate contact with the newborn. In sum, women expressed a desire is to fully enjoy the childbirth experience and pain was viewed as a natural but inevitable part of this process. Although most women desired minimal pain, they also welcomed this sensation as a unique and intrinsic part of being a mother. Participants made a direct and close relationship between VD and severe pain. This was especially true in the public sector where epidural anesthesia is not available for VDs. Sample testimonies included:

I hope that it won’t be so painful, I heard that it is very painful that you cannot resist pain, that it will be the one and only time that I will want to have a baby …” (Woman from Public sector)

“If I can handle the pain, honestly, I wouldn’t ask for anesthesia…I am preparing with breathing exercises, trying to incorporate all I’ve learned, in yoga and at the gym, about how to calm yourself.” (Woman from non-public sector)

Non-public sector women can choose to have anesthesia and often preferred epidural anesthesia to control pain; however most women preferred only mild anesthesia in which there was a reduction in pain but where they could still feel contractions and push.

“There is no need to suffer like crazy if you can have anesthesia.” (Woman from non-public sector)

“I would have anesthesia as long as I do not lose the feeling of the contractions with which you push.” (Woman from non-public sector)

Regardless of use of epidural anesthesia, women from both public and non-public sectors described childbirth pain in positive terms. It was considered a special kind of pain perceived as “*unique,”* “*beautiful,”* “special,” “*linked to life,”* “*natural,”* “*an expected pain*,” and a type of pain which is “*worth suffering*.”

“I told myself, ‘It’s going to hurt.’ But I’m not afraid because it’s a natural pain.” (Woman from Public sector)

In addition, the majority of women perceived pain to be only temporary, and that after the baby is born the woman would immediately forget about the suffering.

“It is a life-learning experience that has to do with passing through this pain more than resisting it.” (Woman from non-public sector)

“Beyond the pain, I know it is a joyful and a unique moment, so I would like to enjoy it the most I can…it shouldn’t be a traumatic moment.” (Woman from Public sector)

### Beliefs about mode of delivery

#### Vaginal Delivery

Psychosocial factors surrounding women’s preferences included cultural beliefs. Participants identified several beliefs about the advantages of VD--it is viewed as “natural,” provides satisfaction, and viewed as a rite of passage into motherhood.

“Vaginal delivery is natural. Women have delivered babies like that since the beginning of the history of humankind. Other forms of delivery are man’s inventions.” (Woman from non-public sector)

“Vaginal delivery allows women to ‘feel’ the process of living birth, an experience that will be remembered forever.” (Woman from Public sector)

“Satisfaction is bigger, since you have made the effort. Emotionally it cheers you up.” (Woman from Public sector)

“Giving birth naturally is part of being a mother.” (Woman from Public sector)

“In a vaginal delivery the baby takes part in the delivery process, it pushes with the mother and this favors a good mother-baby relationship.” (Woman from Public sector)

Women also highlighted practical concerns regarding caring for the baby immediately following childbirth related to preferences.

“Recovery is faster so women can take better care of the baby after a vaginal delivery.” (Woman from non-public sector)

“Women can breastfeed immediately after a normal delivery.” (Woman from Public sector)

Some women also believed that women have more milk after VD than after a CS; and that as the baby participates in the delivery process it is born hungry and thus breastfeeds easily.

Most disadvantages of VD focused on practical concerns, in particular those related to physical or medical factors, including long hours of labor, prolonged pain, exhaustion, and episiotomy stitches that might be uncomfortable for some days after delivery. Women expressed concern for future health problems like prolapse, vaginal lacerations, and incontinence as a result of VD.

Only one participant from the non-public sector mentioned that the size and shape of the vagina can change after delivery affecting sexual life; however this was not identified as a reason to reject vaginal delivery or to prefer CS.

Many women also focused on psychological stress, including anxiety and fear of the unknown. Although in some circumstances, CS was viewed as providing more control specifically of the timing and pain, it was also viewed as providing less control. Because the CS was viewed as primarily a medical procedure indicated only when there were complications, a VD provided a greater sense of control that the delivery was going “naturally” or “as expected.”

“There are higher chances of unpredictable events in vaginal delivery than in CS where everything seems to be more under control. If complications arise during a vaginal delivery the woman might need to undergo an emergency caesarean section with possible risks for herself and the baby.” (Woman from non-public sector)

Uncertainty surrounding the timing of delivery was of particular concern. Women worried that they might be tired when labor starts or that they will be away from their homes or even far away from the hospital where they plan to deliver. Some women worried that labor may start earlier than the expected delivery date. For example, right before the delivery date, women may experience “false alarms,” such as contractions. As a result, she may go to the hospital earlier than expected but be turned away because she is not ready to give birth. Others worried that they might not be prepared, such as have everything ready to go to the hospital (e.g., the bag with their clothes and the baby’s clothes) or that they might not have finished the arrangements at home to receive the baby.

Some women expressed fear of not knowing what to do when it is time for the delivery. They expressed fear that they might not know how to push or that they might push incorrectly. Women wondered about whether they would have the “maternal instinct” that will naturally guide them through childbirth.

#### Caesarean Section

Most of the participants stated that they preferred to deliver vaginally. Therefore, CS was perceived primarily as a medical decision based on the health condition of the woman or infant. CS was most often associated with intrapartum emergency situations (e.g., umbilical cord twist), the mother’s health problems (e.g., hypertension or sexually transmitted infections), the baby’s position, or other problems associated with VD (e.g., lack of dilation, not being strong enough to push, post-term pregnancy). CS was rarely viewed as an elective option that they woman would specifically choose.

*“From my point of view, cesarean does not have any benefits. It is more like a solution, I would just prefer a cesarean if delivery gets complicated”* (*Interview participant from non-public sector)*

When probed to identify the advantages for CS, most were able to identify a few. The most frequently cited perceived advantages included the rapidness of the procedure, limited pain at the time of childbirth, and less traumatic for the baby (e.g., cyanosis). Convenience was also discussed, especially about having the baby on a fixed date. Many women reported that this would help them feel less anxious, as the events were viewed as predictable with limited uncertainty about the date of delivery and the limited possibility of false alarms or repeated pelvic exams. Despite this, the general view of the women was that CS was viewed as a procedure indicated in order to reduce risk in the case of unfavorable health conditions.

Women also identified several disadvantages of CS. Regarding cultural and psychosocial factors, many women stated that during a CS, women have no active role, are asleep and therefore are not able to “*feel childbirth.*” For this reason, CS was viewed as a less emotionally moving experience and some participants stated the belief that not being able to have a VD could be a cause of depression for women.

Women also identified several practical disadvantages of delivery by CS. These included a slow recovery, greater pain, risks associated with any surgical procedure, that are similar to any other type of surgery, loss of autonomy during recovery, and delayed interaction with the infant. Anesthesia was a major concern due to possible complications. Women also feared that the anesthesia might not work properly or be incorrectly administered and as a result, they would feel pain during the procedure. Women also stated that with anesthesia they might feel “*sleepy*”, “*dizzy*” and with “*loss of awareness,*” which might prevent them from taking care of the baby.

“*If the anesthesia is not correctly administered your body can be paralyzed.*” (*Woman from Public sector)*

Participants stated the belief that after a CS, women would be unable to take pain medications because it may pass to the breast milk and therefore affect the baby’s health. There is a widespread belief among the sample that once a woman has had a CS, all future deliveries will have to be by CS. This would mean that she would not be able to have “*normal”* deliveries in the future, something that was highly valued by the majority of our participants.

Additionally, the scar left by a CS was viewed as a disadvantage depending on the size and type of the incision and the healing process. Most women however, agreed that modern CS techniques involve minor incisions that leave only small scars.

Family roles and responsibilities were also identified as major disadvantages to a CS, including that a spouse or partner cannot always be present during this procedure. After delivery, the newborn becomes the priority for the family and the caretakers. In this environment women do not get enough rest or adequate post-surgery care. Moreover, the additional need for assistance from a spouse or family member to take care of both the baby and herself (walking, bathing, breastfeeding) was viewed as a major disadvantage.

The women endorsed several beliefs about delivery by CS, including that they might have less breast milk after a CS and that they might have to wait longer to get pregnant again. Some women also viewed a CS as more traumatic for the baby since the delivery is abrupt compared to a VD; others viewed a VD as more traumatic because the baby has to struggle during the childbirth process with possible risk of cyanosis, whereas the CS was more rapid.

### Sources of information on pregnancy and delivery

Regarding sources of these beliefs, family and friends were identified as an important source of information. Women did not report their partners making a significant contribution to their decision-making for mode of delivery.

This was at times described as confusing as they might receive different or contradictory messages from friends and family members:

“Your grandmother tells you to do this, your aunt tells you to do that…” (Woman from Public sector)

“My aunt had a vaginal delivery and a cesarean; she had 2. She preferred the cesarean…but just yesterday I was talking with my grandmother and sister-in-law and between vaginal delivery and cesarean, they preferred vaginal.” (Woman from Public sector)

“I go to my grandmother, because she always gives me advice.” (Woman from non-public sector)

Books, popular media and prenatal courses were another source of information. Although many of the non-public care women had read books and taken prenatal courses, they still wondered whether they would be able to practice what they have learned, particularly considering that during delivery, pain can prevent them from concentrating on the breathing and relaxing techniques they learned.

Public health sector users were generally younger than non-public sector users. They identified family and friends as the main sources of information of pregnancy and delivery. They also gave importance to television programs.

Non-public sector women mentioned a wider variety of sources including books, videos, and the Internet, as well as family and friends.

The most important difference in sources of information between non-public and public users was the relationship they had with their health providers. Non-public sector women had built stronger rapport with their providers and as a result, were able to ask questions and gain more information from them. In contrast, public sector women did not feel as strong a connection with their providers. Public sector pregnant women did not establish strong rapport with their health providers because they are seen by a variety of providers during prenatal care often in very busy contexts. Consequently, women did not feel comfortable asking questions or requesting information. In addition, most of them completed their prenatal check-ups at primary health care centers with midwives, and are later referred to the hospital for delivery.

### Choosing mode of delivery

For most public sector women, the mode of delivery was not a choice but a medical decision. Meanwhile, with non-public sectors women, the mode of delivery was sort of a mutual decision with their professionals.

Even though most women did prefer a vaginal delivery, women from the non-public sector arrived at this decision themselves, whereas those from the public sector often had the decision made for them by their physician. That is, non-public sector women took a more active role in the decision, whereas public sector women took a more passive role in the decision.

“… I think that you kind of give yourself up to your doctor, and if he says, “lay down and open your legs” you will do it. You would never reply that you want to deliver “crouching or standing”…. conditions are not given for women to choose, because it has to do with regulations and structures. It is not possible to have a physician waiting for you.” (Woman from Public sector)

*“…now it is possible to choose if you want a CS, but me and my doctor have agreed that if nothing comes up, I will have a vaginal delivery.” (Woman from non-public sector*)

“*The doctor decides; he knows better than I do.” (Woman from non-public sector)*

Women from the non-public sector did not feel that they needed to justify a personal choice for CS in the absence of medical indication for this mode of delivery, and neither those from the public sector if they if they had a choice to decide.

For the participants, the reasons that would motivate women to request an elective CS included beliefs that the women were driven by an exaggerated fear of childbirth and pain and by the perception that they would not be capable of having a normal delivery. The participants also agreed that the provider’s decision to perform a CS due to medical reasons would not be questioned.

Non-public sector women expressed concern about unnecessary CS performed with the purpose of rushing deliveries in order to vacate hospital beds to allow new admissions. Participants emphasized the importance of being careful in choosing a doctor, looking for someone who shares the woman’s *“ideology”* and “*style”* and establishing a good relationship with him/her. Once the woman is sure that she has chosen the right provider, then she would be able to trust that all decisions would be based on medical criteria respecting her preferences.

On the other hand, public sector women did not have the option of choosing their doctor and often expressed mistrust towards their providers due to poor quality of care and a widespread notion that nurses and doctors often underestimate primigravida women’s pain or suffering. Participants shared stories of women whose complaints and requests were ignored and this eventually led to complications and at times, permanent harm to the baby or mother.

“It depends on the hospital where you’re going to have the baby or even the country too.” (Woman from Public sector)

“In this hospital, I don’t think you can choose…it depends on the doctors.” (Woman from Public sector)

## Discussion

In this study, we found that most women prefer vaginal delivery. Reasons cited include those that are cultural, personal, and social. Vaginal delivery was viewed as normal, healthy, and a natural rite of passage from womanhood to motherhood. Contrary to reports suggesting that women fear pain associated with childbirth [9-12,14,15,19] pain associated with VD was viewed in a positive light. In contrast, women viewed CS as a medical decision and often defer decisions to medical staff in the presence of medical indications for CS.

A major strength of our study was use of qualitative research to investigate the influence of obstetric and psychosocial factors on women’s views of vaginal and cesarean birth. As a result, we were able to draw conclusions about what women view as important factors that affect their decision and their perspectives on vaginal delivery versus CS, including the advantages and disadvantages of both. We were able to compare differences between the public and non-public sectors of obstetric care and consider how relationships with physicians and midwives and access to information affect women’s preferences for mode of delivery. One limitation is that our study only targeted nulliparous women and so the results may not apply to all pregnant women. It is possible that multiparous women, with 1 or more previous vaginal deliveries or cesarean sections, could have different perceptions and preferences for route of delivery based on their own personal experience and be less influenced by cultural and social factors. A comparison of the preferences of multiparous and nulliparous women should be an important topic for future research, to determine how perceptions of both groups contribute to the high CS rates worldwide. We only performed 4 FGDs and 12 in depth interviews, which may have limited the diversity of perspectives and points of view about women’s preferences for mode of delivery. Additionally, our results are derived from a sample of women in Buenos Aires, Argentina; however our results may be transferable to similar situations or contexts (e.g., with Latin-American women) where these findings may be relevant and applicable.

Nevertheless, our findings converge with existing research and shed important light about women’s preferences. A recent systematic review about women’s preferences for cesarean section included thirty-eight studies of 19,403 women, found that the overall preference for caesarean section was only 15.6% and that most women prefer vaginal delivery [20], which is consistent with our results. Our findings extend this literature by showing that women have a strong preference for vaginal delivery, which they consider natural, human, beautiful, and the “real experience” of becoming a mother.

## Conclusions

In our study, we found that actual CS rates appear incongruent with the women’s preferred mode of delivery, VD. These findings converge with quantitative and qualitative studies showing that women are highly inclined towards vaginal delivery for various cultural, personal and social factors [20]. Cesarean section rates in many middle and high-income countries are increasing and it may not be due to women’s preferences alone. Using FGDs, we were able to determine several cultural, personal and social factors play an important role in nulliparous women’s preferences. Studies of interventions aimed at reducing cesarean section rates should assess women’s preferences using a standardized measure to examine additional factors that might be driving the high cesarean section rates.

## Competing interests

The authors declare that they have no competing interests.

## Authors’ contribution

NHL, AM, FA and JMB conceived and designed the study. NHL, NZ, LA, and OHC collected the data. NZ, NHL and MC analyzed the data. NHL, AM, JMB, MC, NZ, OHC, and LA interpreted the data. NHL drafted the manuscript in collaboration with AM, NZ, MC, FA and JMB. All authors read and approved the final manuscript.
